# Highly homologous eEF1A1 and eEF1A2 exhibit differential post-translational modification with significant enrichment around localised sites of sequence variation

**DOI:** 10.1186/1745-6150-8-29

**Published:** 2013-11-13

**Authors:** Dinesh C Soares, Catherine M Abbott

**Affiliations:** 1 MRC Human Genetics Unit, MRC Institute of Genetics and Molecular Medicine, University of Edinburgh, Western General Hospital, Crewe Road South, Edinburgh EH4 2XU, UK; 2 Centre for Genomic and Experimental Medicine (CGEM), MRC Institute of Genetics and Molecular Medicine, University of Edinburgh, Western General Hospital, Crewe Road South, Edinburgh EH4 2XU, UK

**Keywords:** eEF1A1, eEF1A2, Phosphorylation, Methylation, Acetylation, Ubiquitination, Post-translational modification

## Abstract

Translation elongation factors eEF1A1 and eEF1A2 are 92% identical but exhibit non-overlapping expression patterns. While the two proteins are predicted to have similar tertiary structures, it is notable that the minor variations between their sequences are highly localised within their modelled structures. We used recently available high-throughput “omics” data to assess the spatial location of post-translational modifications and discovered that they are highly enriched on those surface regions of the protein that correspond to the clusters of sequence variation. This observation suggests how these two isoforms could be differentially regulated allowing them to perform distinct functions.

**Reviewers:**

This article was reviewed by Frank Eisenhaber and Ramanathan Sowdhamini.

## Background

Translation is the mechanism by which the cell accomplishes *de novo* protein synthesis. The elongation stage is where aminoacylated-tRNAs (aa-tRNA) are delivered to the ribosome, is accomplished by elongation factor 1A, or eEF1A, which is the second most abundant protein in the cell; the role of eEF1A is GTP dependent, and this process is facilitated by a GTP-exchange factor called eEF1B. In vertebrates, eEF1A occurs in one of two different isoforms (eEF1A1 and eEF1A2) each encoded by a separate locus, and each with a distinct expression pattern [1,2]. These two isoforms are 92% identical and 98% similar to each other at the amino acid level. eEF1A1 is almost ubiquitously expressed except in neurons and muscle (skeletal and cardiac), where eEF1A1 declines to undetectable levels and is gradually replaced by eEF1A2 during postnatal development [3]. This expression pattern correlates perfectly with the onset of neuromuscular abnormalities in wasted mice [3]. Biochemically, the two isoforms appear to operate in a similar manner; they have indistinguishable activities in an *in vitro* translation system, but eEF1A2 shows much greater affinity for GDP than GTP, whereas eEF1A1 has a greater affinity for GTP [4]. We recently used comparative homology modelling to map those amino acids that differ between the two isoforms onto their tertiary structures. This revealed that the non-conserved residues appear in discrete surface clusters that do not overlap with the binding site for eEF1Balpha [5].

A crucial question is why eEF1A1, which is highly conserved throughout evolution, widely expressed, and is an abundant and essential protein, should be switched off in certain tissues at specific developmental stages and replaced with an almost, but not quite, identical and equally highly conserved protein? One possible explanation is that eEF1A1 has additional “moonlighting” or non-canonical, functions that may not be shared by eEF1A2, and that might in fact be deleterious to certain cell types.

What are these non-canonical functions? It has been demonstrated that eEF1A1 can interact with and modify the cytoskeleton [6]. If on the other hand eEF1A2 did not share these properties, this would fit well with the nature of many of the cell types that we know undergo this switch - they tend to be cells with a very stable and complex architecture. However, there are now many other examples of non-canonical functions for eEF1A1, few of which have been investigated with respect to eEF1A2. Roles have also been identified for eEF1A1 in viral propagation, proteolysis, nuclear transport and apoptosis [7], but it is important to note that some of these roles may not be truly non-canonical, in that they may not be independent of the role of eEF1A1 in translation; one of the challenges for the field is to start to address these subtleties. eEF1A1 has been found to be crucial for the function of many viruses [8] whereas eEF1A2 is not known to fulfil a similar role. eEF1A2 is a putative oncogene; it has been shown to be capable of transforming cells that are already expressing high levels of eEF1A1, suggesting that the two proteins may have different roles in tumourigenesis [9]. Disentangling the non-canonical functions with respect to each of the two isoforms is crucial because of the distinct roles they play in disease.

## Findings

**Figure 1:**
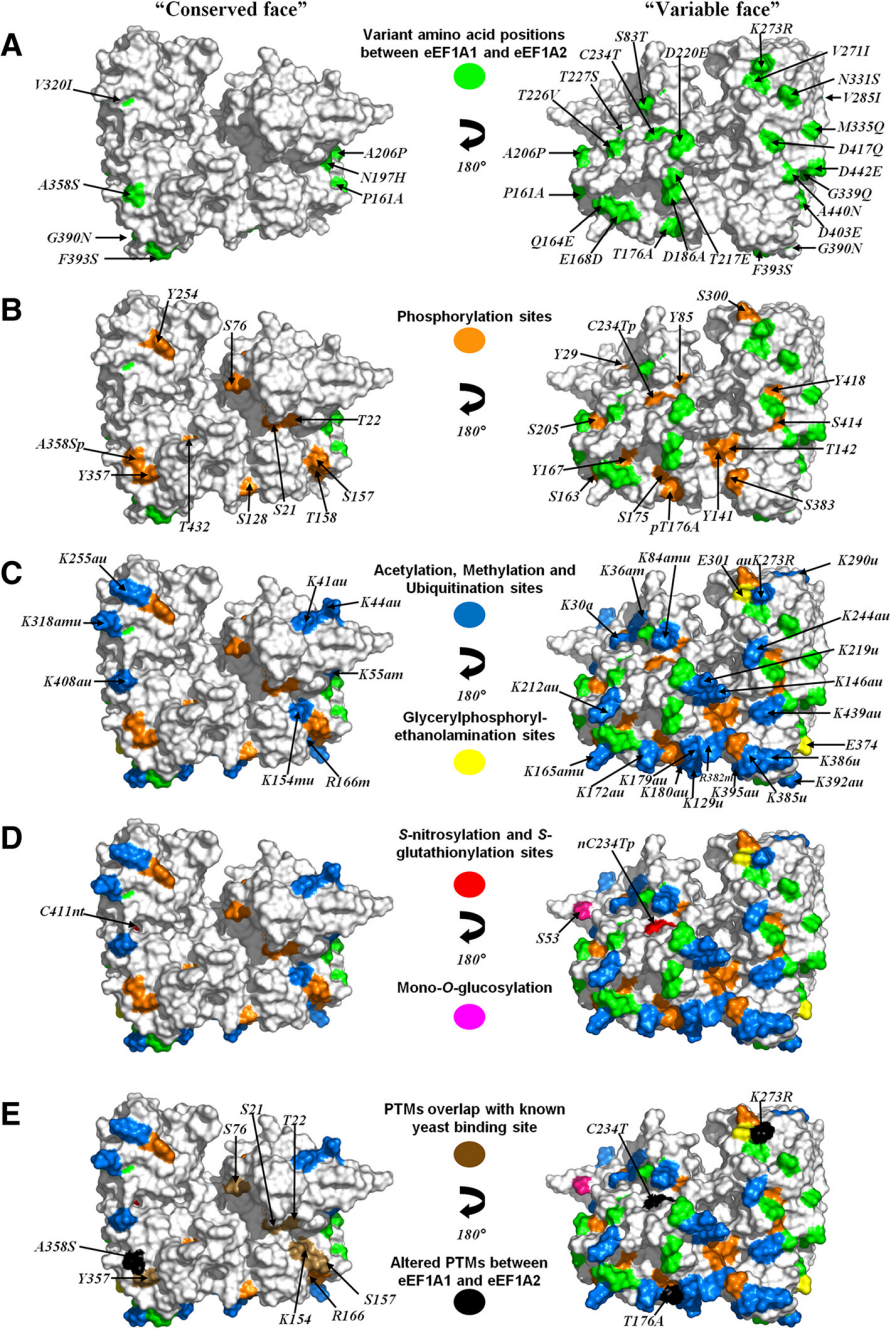
**Post-translational modification and eEF1A1 and eEF1A2.** All known PTMs are mapped on the surface of the 3-D model of eEF1A1 are shown in the two views (the “conserved face” and the “variable face”) rotated by 180° about the *y*-axis: **(A)** location of variant amino acids between eEF1A1 and eEF1A2 (green); **(B)** phosphorylation sites (orange); **(C)** acetylation (blue), methylation (blue), ubiquitination (blue) and ethanolamination (yellow) sites; **(D)***S*-nitrosylation (red), *S*-glutathionylation (red) and *O*-glucosylation sites (pink); **(E)** overlap of post-translational modifications with known binding sites (brown) and altered PTMs between eEF1A1 and eEF1A2 (black). All surface-exposed variant amino acid residues between eEF1A1 and eEF1A2 and PTMs are labelled; where a residue is modified in more than one way or altered between the two isoforms, this is indicated on the residue (a = acetylated; m = methylated; n = *S*-nitrosylated; p = phosphorylated; t = *S*-glutathionylated; u = ubiquitinated).

We previously mapped known and variable (potential) phosphorylation sites on comparative models of eEF1A1 and eEF1A2. Since that report, high-throughput “omics” studies using tandem mass spectrometry have emerged that have uncovered additional novel phosphorylation sites, as well as sites of acetylation, methylation and ubiquitination. Based on literature and the PhosphoSitePlus database [10], we have now mapped these new data to our models. This exercise revealed interesting differences in post-translational modifications (PTMs) between the two variants. These include examples of altered acetylation, phosphorylation, *S*-nitrosylation and ubiquitination that can only occur in one of the variants. These are shown mapped on a modelled structure in Figure 1 (and on a multiple sequence alignment in Additional file 1; tabulated in Additional file 2). Specifically, T176A can only be phosphorylated in eEF1A1, C234T can be *S*-nitrosylated only in eEF1A1 but can be phosphorylated in eEF1A2, K273R can only be acetylated and ubiquitinated in eEF1A1. More recently one of our predicted sites of phosphorylation, S358A that is specific to eEF1A2 [5], was experimentally confirmed [11].

There are 74 post-translationally modified positions in total. Seven of these are located within the C-terminal disordered region of the protein [5], while a total of 67 sites can be mapped onto the 3-D model of eEF1A1 for analysis. Out of these 67 sites, 56 modified residues are largely or partly exposed on the surface of the protein (Figure 1). The vast majority of PTMs map on to the “variable face” of the protein and many lie either within or close to the two clusters of sequence variation between eEF1A1 and eEF1A2 (Additional file 3). The “variable face” has a total of 37 modified residues, compared with just 19 such residues seen on the “conserved face” of the protein – an almost two-fold increase. A total of 24 sites are modified by more than one receptor implying competition between modifying enzymes (Additional file 1). Taking into consideration residues that can be alternatively modified the “conserved face” displays 28 possible modifications amongst 19 residues, while the “variable face” supports a total of 53 modifications. This observation is surprising considering the number of PTMs present. Figure 1 has been systematically redrawn in each panel with the new PTM shown on the surface where they overlap to aid the viewer to distinguish between different PTM-types.

Some of the PTMs overlap with known binding sites from experiments conducted in yeast eEF1A (Figure 1, Panel E). These include S21, T22, K154, S157 (GTP/GDP-binding site), S76 (eEF1Balpha-binding site; note Alanine (A76) is present at this equivalent position in yeast), R166 (mutant reduces dependence on eEF1B), N331S, M335Q and Y357 (mutants involved in actin bundling/organisation). Interestingly, there are only two residues (24 out of 26 are strictly conserved) that are altered between yeast and human eEF1As for the eEF1Balpha-binding site. These are: S76 in human, A76 in yeast; and I89 in human, V89 in yeast. The latter is a conservative substitution, but the observation that S76 is apparently phosphorylated in human eEF1A but not in yeast may thus suggest differential binding or regulation with eEF1Balpha. The effect of this single amino acid substitution in the human eEF1A1-eEF1Balpha interaction should be tested. There already exists evidence for post-translational modulation of function for the eEF1As; phosphorylation at S300 by TGFbetaR-1 was shown to prevent aa-tRNA binding to eEF1A1 [12] (Figure 1, Panel B), and mono-*O*-glucosylation at S53 in eEF1A is known to inhibit protein synthesis [13] (Figure 1, Panel D).

Another notable observation is that the “base” (see variable-face view) of the molecule is very highly enriched for lysine and arginine modification (acetylation, methylation, ubiquitination). There is also another surface on the “left” (see conserved-face view) that forms an extended binding region for the modifying molecules. Analysis of our previously undertaken electrostatic surface mapping on the eEF1A1 and eEF1A2 model molecules [5] in this context reveals some important changes. These involve charged amino acids that occur in immediate proximity to acetylation, methylation and ubiquitination sites only on the “variable face” of the molecules. Specifically, these are D186A and D417Q - negative charge in eEF1A1, absent in eEF1A2, and Q164E and T217E - negative-charge in eEF1A2, absent in eEF1A1. These could impart different molecular recognition properties to the modifying molecule.

Not all of the post-translationally modified residues are surface exposed (a total of 11 out of 67 are completely buried), indicating that the protein adopts different conformations depending on its bound *versus* unbound state (for example with eEF1B) that reveal or conceal sites targeted by the modifying proteins. It is possible that local conformational changes or structural rearrangements may underlie greater surface accessibility for some of these buried sites [14]. Moreover, the active site of the protein-modifying enzyme needs to access the target residue on the substrate; this situation could be made more likely if the substrate existed in a less compact state or if modification occurred prior to protein folding [15,16]. Indeed, a previous study by Budkevich et al. [17] provided evidence that the native eEF1A1 isoform possesses a non-globular extended conformation in solution that changes to a more compact conformation upon interaction with tRNAs.

An example of a divergent, functionally important patch is the region that contains the ethanolamination site E301 [18,19], the competitive (altered) acetylation and ubiquitination site K273R, the phosphorylated site S300 (known to prevent aa-tRNA binding) [12,20] and two variants between eEF1A1 and eEF1A2 (N331S and M335Q, Figure 1 “variable face”) that impact on actin binding and bundling properties (from yeast mutagenesis data [21,22] – summarised on structure in Soares et al. 2009 [5]). Additional comparative sequence analysis, of vertebrate eEF1A1 and eEF1A2 with yeast eEF1A, reveals clear evidence for alterations in post-translational modifications between them (Additional file 1). Hence, the presence of the two distinct cell type-specific isoforms in vertebrates creates the potential for greater functional complexity than is seen in yeast.

We mapped all known PTM data on the 3-D models of eEF1A1 and eEF1A2. We note that while the PhosphoSitePlus resource is based upon experimental, manually curated data there remains a possibility for false positives, particularly from the large-scale mass spectrometric studies. Experimental verification of PTMs discovered by mass spectrometry will be needed using additional complementary techniques. It is also of note that many of the studies have been performed on human tumour or cancer cell line material; it is therefore conceivable that some of these PTMs may be specific to tumours, depending on the relative expression of different modifying enzymes in cancerous and normal cells. Additionally, while a large body of PTM data is available for these two proteins (Additional file 2) new sites are still being discovered. For example the aforementioned eEF1A2-specific phosphorylation of S358 [11] was not observed in the high-throughput proteomics screens. Hence, false negatives are also possible given the sensitivity of the methodology [14]. Owing to the very high sequence identity between eEF1A1 and eEF1A2, it is currently almost impossible to judge whether the experimentally identified post-translationally modified peptides originated from one isoform or the other. Because so many PTMs are located proximal to sites of sequence variation on the surface of the protein (Additional file 3), we speculate that the modified residues will in fact often be isoform specific. Owing to the large number of clustered PTMs it is likely that extensive cross-regulation occurs [23-25]. Furthermore, the protein-modifying enzymes involved could differ between the two isoforms, even for sites that are identical in sequence. Examining these possibilities remains a key future goal for research.

In a recent biophysical study that compared eEF1A1 and eEF1A2 [26], the authors noted the main difference in structural properties of these proteins was an enhanced ability of eEF1A1 to self-associate. Thus even though the two proteins are predicted to have similar tertiary structure [5,27], their oligomeric states differ. Furthermore, the authors established that eEF1A1 was more hydrophobic in character than eEF1A2 [26]. The authors postulated that differential phosphorylation may underlie difference in self-association propensity for the two proteins, and differential methylation profiles could explain the increased hydrophobicity of eEF1A1 [26]. We suggest that the surprising enhancement of PTM in the vicinity of surface clusters of sequence variation is unlikely to have occurred by chance. It has likely evolved to facilitate differentially tuneable properties, such as structural or oligomeric propensity and regulation, and thus functional divergence, allowing retention of two non-redundant isoforms.

## Reviewers’ reports

We thank the reviewers for their useful feedback and constructive comments for improvement of our manuscript. We have considered all their suggestions and have addressed these below.

### Reviewer 1: Dr. Frank Eisenhaber (Bioinformatics Institute, Singapore)

In an earlier study, the authors concluded that the minor sequence variations distinguishing eEF1A1 and eEF1A2 are confined in structurally limited sections of the two proteins (mostly at what they call back side). Here, they claim that these places harbour most of the PTMs. A few mutations are suggested that might have an effect on differential binding/regulation.

There are several concerns beyond the issue that the finding might be incremental.

First of all, the authors operate at a quite qualitative level. It would be good to consider in number terms how many PTMs are in the sequence variation clusters, how many are just nearby and how many are differentiated between the two proteins. Together with some statistical assessment, this would substantiate the main claim of this work, namely the enrichment of PTMs in the clusters of sequence variation.

Authors’ Response: The reviewer is right and we thank them for making this point. In the revised text we make clear 1) the total number of PTMs present, 2) how many are surface exposed on each face of the protein, and 3) what residues are subject to more than one form of modification. This quantification substantiates our point that PTMs are significantly more likely in locations close to sites of sequence variation, on the “variable face” of the protein, than elsewhere. From the number-counts there is an almost two-fold increase in modifications on the variable face of the protein (19 on the conserved face; 37 on the variable face). The second paragraph of the Findings section now reports and clarifies these results. We have also added in new Additional file 3: “Post-translational modifications (PTMs) in structural proximity to sequence variants between eEF1A1 and eEF1A2” - to enumerate and specify which PTMs are proximal to each variant using a structure-based 5-Angstrom sphere-radius cut-off for each residue. We have also revised Figure 1 (all PTM residues are now labelled and further annotated if a specific residue can be modified in more than one way) that now clearly emphasises the non-random distribution of PTMs.

Second, the PTMs have been measured in varying biological contexts; some might be artifacts or only applicable to specific biological situations. It would be good to have a separate consideration as detailed above for the PTMs that might be considered most reliable and most likely to be biologically significant.

Authors’ Response: We completely agree with the referee, and have stated the limitations in the text. For example we say: “Experimental verification of PTMs discovered by mass spectrometry will be needed using additional complementary techniques. It is also of note that many of the studies have been performed on human tumour or cancer cell line material; it is therefore conceivable that some of these PTMs may be specific to tumours, depending on the relative expression of different modifying enzymes in cancerous and normal cells.” The PhosphoSitePlus database is a manually curated database of good quality, however most site assignments are not linked with corresponding spectra at present. Therefore, in order to place some measure of reliability on the basis of the quality of evidence we have provided an additional layer of annotation in the revised version (also see reply to Reviewer 2 point 4, for discussion of false positive rates). So for example where a specific experiment has confirmed a particular site in the published literature, and/or a number of five or more citations are associated with a specific site from the high-throughput mass spectrometry screens in PhosphoSitePlus, we have now indicated this in the table in a revised Additional file 2. It is interesting that the majority of high-throughput sites have been repeatedly seen in proteomic screens and thus almost certainly represent true modifications (specifically, for Phosphorylation: 22 out of 36; Acetylation: 11/25; Methylation: 5/9; Ubiquitination: 23/25 have five or more citations assigned to them in PhosphoSitePlus). We have also prepared an additional figure to depict where these more reliable PTM sites are located on the surface by means of a comparison with Figure 1. As is readily apparent in this new figure (Additional file 4), when only the more reliable PTMs are considered, there remains an almost two-fold enhancement of post-translationally modified residues on the variable face (conserved face: 16 modified residues; variable face: 31 modified residues).

Third, there are serious issues to which extent PTMs can occur in globular sections. The problem is that protein-modifying enzymes have active site clefts/cavities and the polypeptide stretch of the substrate protein has to get somehow into it (Eisenhaber et al., Current Protein and Peptide Science, 2007, 8, 197). The problem disappears if there are auto-catalysis, non-enzymatic reactions, modifications prior to substrate protein folding, unstructured segments/long, conformationally variable loops or unstable structural parts that readily unfold. It would be necessary to substantiate what mechanism is going on with eEF1A1/2 since this is part of the proof that the PTM seen is biologically significant.

Authors’ Response: Again, the referee is right to make this point. There are however, several PTMs that have been confirmed by site-specific experiments, which are indeed surface-exposed on more structured regions (e.g. S21, K36, K165 on alpha-helices; E374 on beta-strand etc.). The eEF1A1/eEF1A2 models [5] were based upon the co-crystal structure of (~81% identical) yeast eEF1A when bound to eEF1Balpha [28], so it is hard to speculate on what conformational changes occur when eEF1A transitions from free to bound forms. It is indeed possible that protein-modifying enzymes access their substrates in less globular forms of eEF1A. Some evidence for less globular structure is provided in Budkevich et al. [17] who suggested that the eEF1A1 isoform possesses an extended conformation in solution that changes to an essentially more compact conformation upon interaction with tRNAs. In support of such structural rearrangement, Negrutskii et al. [14] discussed tyrosine phosphorylation in the elongation factors from proteomic studies and noted burial of some of these residues. While we can’t experimentally substantiate context-dependent structural rearrangements in the current paper, it is notable that (as yet) there is no evidence that the eEF1A isoforms are bound to eEF1B (and therefore in this structured conformation) when performing their non-canonical roles in other functional pathways. Other protein-interaction dependent PTM may also influence accessibility of specific residues to their modifying enzymes. In sum, at least with respect to this “structured” conformation of the eEF1As bound to eEF1Balpha, it does seem highly unlikely that the observed clustering of PTMs around sites of sequence variability on one face of the protein has occurred by chance. In response, we have updated the main text so that it covers the substance of the above observations.

Quality of written English: Acceptable.

### Reviewer 2: Dr. Ramanathan Sowdhamini (Tata Institute of Fundamental Research, India)

This manuscript by Soares and Abbott report the comparison of post-translational modification (PTM) data of eEF1A isoforms. The two isoforms under analysis, eEF1A1 and eEF1A2, exhibit differences in tissue localisation and affinity for GTP, despite sharing 91% identity in amino acid sequence. These functional differences have been addressed by the authors in the context of the observed differences in patterns of PTM of the two proteins. I would recommend publication of this manuscript in Biology Direct, after the following points have been addressed:

1. Page 4 onwards: terms like ‘front’ or ‘back’ of the protein sound too colloquial.

Authors’ Response: We have revised all text and figure calls in the manuscript to refer to the previous ‘front’ side of the protein as the ‘conserved face’ and the ‘back’ side as the ‘variable face’ to highlight the eEF1A isoform specific location of the sequence-variation in context of this study.

2. Even in cases where PTM sites are conserved between eEF1A1 and eEF1A2, the molecular players responsible for PTM could be dramatically different. The authors need to consider this point.

Authors’ Response: We thank the reviewer for mentioning this point. Indeed, it is very likely that the protein-modifying enzymes involved could differ between isoforms, even for sites that are identical in sequence. This may be influenced by minor differences in the neighbouring structural landscape of surface charge and hydrophobicity proximal to the modified amino acid residue. We have introduced this point in the penultimate paragraph of the Findings discussion.

3. It will be interesting to compare such PTM motifs of homologues - both closely related and distantly related - to provide a dimension of the role of PTM in the context of evolutionary dynamics.

Authors’ Response: The referee is right. The issue with extending the evolutionary analysis is one of available data. Given the high similarity of the two isoforms within a species, it is important to have some form of additional evidence in order to assign sequences as unequivocally orthologous to either eEF1A1 or eEF1A2. The best evidence comes from expression analysis; if within a species there are two apparent eEF1A sequences and one is ubiquitously expressed but the other expressed only in brain and muscle, it is possible to have more confidence in their identity as eEF1A1 and eEF1A2 orthologues, respectively. Unfortunately such evidence is almost entirely lacking for many species. Our Additional file 1 displayed a multiple sequence alignment of various eEF1A1 (10 species) and eEF1A2 (9 species) orthologues from vertebrates. The modified residue is strictly conserved in almost every case. Taking into account the reviewer’s suggestion we have now added into the alignment the more divergent sequences of zebra fish and also of non-vertebrate, but well characterised, yeast eEF1A. This comparison illustrates clear alterations in post-translational modifications between yeast compared with other eukaryotic vertebrates – 18 sites in total. These are indicated in the revised Additional file 1. The presence of the two isoforms in vertebrates creates the potential for greater complexity than is seen in yeast: *Saccharomyces cerevisiae* has two genes encoding eEF1A [29] and *Schizosaccharomyces pombe* has three [30], but the encoded proteins are identical within a given species. We thank the reviewer for this suggestion which clearly points to gain of PTMs among vertebrates across evolution. We have summarised this point in the main text along with the revised Additional file 1 and associated legend.

4. Page 6 - It will be important to identify PTMs recorded for other proteins to note if there maybe false positives.

Authors’ Response: Unless comprehensive complementary experimental validation of high-throughput mass spectrometry studies are undertaken for the two eEF1A isoforms and other proteins it will be difficult to estimate false positive rates. Comparing examples of other proteins as a means of assessing the likelihood of false positives, depends on how that particular protein has been researched. For example, for the comparatively better-studied tumour suppressor protein, p53, a wide-range of PTMs have been confirmed by site-specific methods, which when compared to the mass spectrometry (MS) screens in PhosphoSitePlus (http://www.phosphosite.org/proteinAction.do?id=465&showAllSites=true; accessed 28^th^ October, 2013) show that 95 modified positions exist in total across species, of which 80 were confirmed by site-specific methods and 53 were seen by MS; of the 53 observed by MS, only 15 have yet to be confirmed by a site-specific method. This indicates, at least in this example, a very low false-positive rate; bearing in mind that the rest of the MS sites are yet to be verified independently. The p53 polypeptide is smaller than eEF1As (p53, 393aa *cf.* 462/463aa for eEF1A1/eEF1A2) but has a greater number of known and experimentally verified PTMs. One of us recently published a study on two highly similar paralogous proteins - NDE1 and NDEL1 - that possess similar structures [31] but are differentially regulated post-translationally [32]. In that case, nine out of ten sites verified by non-mass spectrometry experiments in the primary literature for NDE1 and NDEL1 were seen in the MS assignments in the PhosphoSitePlus database. This suggests a high true positive rate; additionally, >50% of sites assigned only using the MS/high-throughput proteomics criteria had five or more citations corresponding to each site in the database, indicating a high potential for other sites to be true positives too. There were also instances where experimentally confirmed sites had less than five associated MS citations.

As mentioned in the main text and in our response to Reviewer 1, because a lot of the data is based upon publicly available high-throughput mass spectrometry data, the assignments are probabilistic by nature and need to be further confirmed experimentally by complementary techniques such as site-directed mutagenesis, phospho-specific antibodies and dominant-negative constructs. Furthermore, future work should aim to ascertain the specific kinases and other modifying molecular players. Nonetheless, our structure-based mapping of PTMs in context of sequence variation of the two eEF1A isoforms and their putative binding sites form the baseline from which future studies will continue to inform on the regulation of these proteins.

Quality of written English: Acceptable.

## Abbreviations

aa-tRNA: Aminoacylated-tRNA; eEF1A1: Eukaryotic translation elongation factor 1 alpha 1; eEF1A2: Eukaryotic translation elongation factor 1 alpha 2; PTM: Post-translational modification.

## Competing interests

The authors declare that they have no competing interests.

## Authors’ contributions

DCS and CMA conceived the study. DCS carried out the analysis. DCS and CMA drafted the manuscript. Both authors read and approved the final manuscript.

## Supplementary Material

Additional file 1 **Alignment of eEF1A1 and eEF1A2 vertebrate orthologues and yeast eEF1A with known PTMs mapped.** Multiple sequence alignment of vertebrate eEF1A1 (red box) and eEF1A2 (blue box) is shown with strictly conserved residue positions depicted with a black background and conservatively substituted or variable positions with a white background [5]. The more divergent yeast eEF1A sequence is also shown aligned below with sequence conservation depicted relative to the vertebrate multiple sequence alignment; only those positions that vary between yeast and the other sequences are shown with a white background on the yeast sequence. Those PTM sites that are not conserved in yeast are indicated with a solid-filled light blue rectangle. The known PTM sites specific to one of eEF1A1 or eEF1A2 are shown within a yellow box (T176A; C234T; K273R; S358A). The location of each PTM is denoted by a symbol above the alignment block: phosphorylation: P; acetylation: A; ubiquitination: U; methylation: M; ethanolamination: E; mono-*O*-glucosylation: G; *S*-nitrosylation: N; *S*-glutathionylation: T; carbonylated peptide: C; where more than one modification occurs at a position this is shown separated by a ‘slash’ in a smaller font. Click here for file

Additional file 2 **Table of post-translational modifications (PTMs) in eEF1A1 and eEF1A2.** All experimentally derived, curated PTMs in the eukaryotic translation elongation factors 1A1 and 1A2 from human, mouse, rat, and rabbit are provided with corresponding references. The list derives from curated information in the PhosphoSitePlus (http://www.phosphosite.org; accessed August 2013) and UniProt databases [10,33], literature involving specific PTM studies in eEF1As, and from both low-throughput experimental methods and high-throughput tandem mass spectrometry. Where data were obtained from mass spectrometric studies from MS assignments from the Cell Signaling Technology research group, the curated results from the PhosphoSitePlus team are listed and referenced. High probability sites represented by five or more references in the PhosphoSitePlus database or those confirmed by experimentation are highlighted in blue (phosphorylation: 22/36; acetylation: 11/25; methylation: 5/9; ubiquitination: 23/25 have five or more citations assigned to them in PhosphoSitePlus); these are mapped on the surface of the 3-D model of eEF1A1 in Additional file 4. Amino acid substitutions that are specific to eEF1A1 or eEF1A2 and that impact on PTMs are highlighted in bold and a short description of the substitution is provided where required. Click here for file

Additional file 3 **Post-translational modifications (PTMs) in structural proximity to sequence variants between eEF1A1 and eEF1A2.** A total of 42 out of 67 (62.7%) PTM sites are located structurally-proximal to a variant amino acid residue between eEF1A1 and eEF1A2; assessed using a 5-Å sphere radius probe measurement around each variant amino acid on the 3-D model of human eEF1A1 [5] under PyMol (http://www.pymol.org; The PyMOL Molecular Graphics System, Version 0.99 Schrödinger, LLC). Those variants that are altered between human eEF1A1 and eEF1A2 and are also sites of post-translational modification are highlighted in bold. Note; there are an additional seven PTM sites located in the unstructured C-terminus of the proteins that harbour four additional sites of sequence variation between eEF1A1 and eEF1A2, not shown. Out of the 42 PTMs located proximal to the variant amino acid residues, a total of 33 are surface exposed at or proximal to the two surface clusters of sequence variation (a total of 20 for cluster 1, and 13 for cluster 2). Twelve of these cluster-proximal PTM sites are modified more than once. Symbols added at the end of each PTM in the table indicate: –p: phosphorylation; -a: acetylation; -m: methylation; -u: ubiquitination; -e: glycerylphosphorylethanolamination; -n: *S*-nitrosylation; -t: *S*-glutathionylation. Click here for file

Additional file 4 **Post-translational modifications (PTMs) by strength of evidence mapped on surface of eEF1A1.** (A) All experimentally derived, curated PTMs in the eukaryotic translation elongation factors 1A1 and 1A2 from human, mouse, rat, and rabbit are shown mapped on the 3-D model of eEF1A1 as in Figure 1 in the main text; refer to legend therein. (B) Only those sites represented by five or more citations in the PhosphoSitePlus database or those confirmed by site-specific experiment in the literature are shown on the 3-D model of human eEF1A1. PTM residues that do not meet this criterion are labelled in the upper frame (A) for comparison. The vast majority of surface exposed residues that harbour PTMs are retained. Click here for file
