# Supplementary figures and images for: Highly homologous eEF1A1 and eEF1A2 exhibit differential post-translational modification with significant enrichment around localised sites of sequence variation
Source: Biol Direct. 2013 Nov 13;8:29. doi: 10.1186/1745-6150-8-29 (PMC3868327; doi:10.1186/1745-6150-8-29)

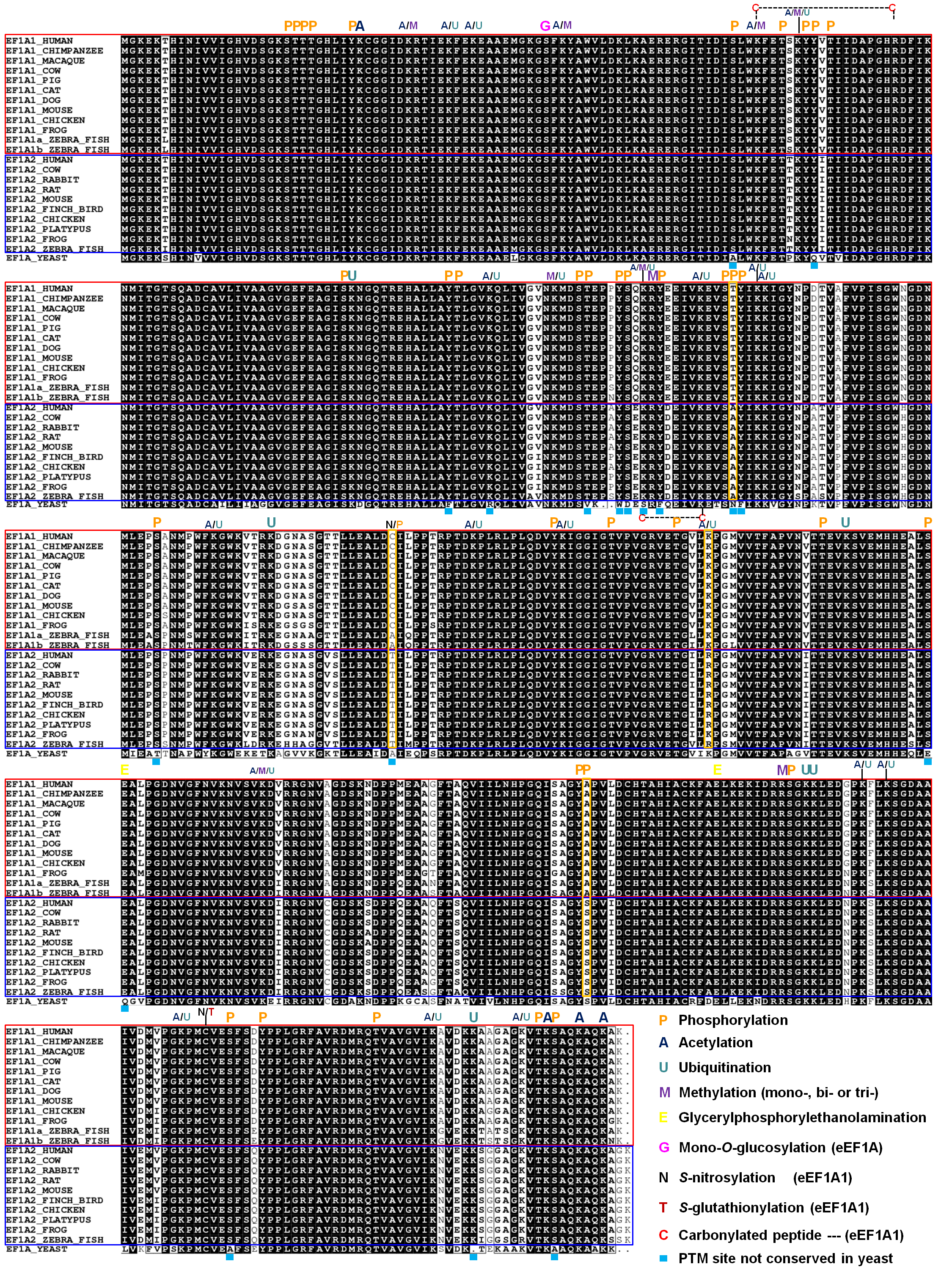

Supplement: Additional file 1 — Alignment of eEF1A1 and eEF1A2 vertebrate orthologues and yeast eEF1A with known PTMs mapped. Multiple sequence alignment of vertebrate eEF1A1 (red box) and eEF1A2 (blue box) is shown with strictly conserved residue positions depicted with a black background and conservatively substituted or variable positions with a white background [5]. The more divergent yeast eEF1A sequence is also shown aligned below with sequence conservation depicted relative to the vertebrate multiple sequence alignment; only those positions that vary between yeast and the other sequences are shown with a white background on the yeast sequence. Those PTM sites that are not conserved in yeast are indicated with a solid-filled light blue rectangle. The known PTM sites specific to one of eEF1A1 or eEF1A2 are shown within a yellow box (T176A; C234T; K273R; S358A). The location of each PTM is denoted by a symbol above the alignment block: phosphorylation: P; acetylation: A; ubiquitination: U; methylation: M; ethanolamination: E; mono-O-glucosylation: G; S-nitrosylation: N; S-glutathionylation: T; carbonylated peptide: C; where more than one modification occurs at a position this is shown separated by a ‘slash’ in a smaller font. [file 1745-6150-8-29-S1.tiff]
