# Supplementary material for: Highly homologous eEF1A1 and eEF1A2 exhibit differential post-translational modification with significant enrichment around localised sites of sequence variation
Source: Biol Direct. 2013 Nov 13;8:29. doi: 10.1186/1745-6150-8-29 (PMC3868327; doi:10.1186/1745-6150-8-29)
Supplement: Additional file 2 — Table of post-translational modifications (PTMs) in eEF1A1 and eEF1A2. All experimentally derived, curated PTMs in the eukaryotic translation elongation factors 1A1 and 1A2 from human, mouse, rat, and rabbit are provided with corresponding references. The list derives from curated information in the PhosphoSitePlus (http://www.phosphosite.org; accessed August 2013) and UniProt databases [10,33], literature involving specific PTM studies in eEF1As, and from both low-throughput experimental methods and high-throughput tandem mass spectrometry. Where data were obtained from mass spectrometric studies from MS assignments from the Cell Signaling Technology research group, the curated results from the PhosphoSitePlus team are listed and referenced. High probability sites represented by five or more references in the PhosphoSitePlus database or those confirmed by experimentation are highlighted in blue (phosphorylation: 22/36; acetylation: 11/25; methylation: 5/9; ubiquitination: 23/25 have five or more citations assigned to them in PhosphoSitePlus); these are mapped on the surface of the 3-D model of eEF1A1 in Additional file 4. Amino acid substitutions that are specific to eEF1A1 or eEF1A2 and that impact on PTMs are highlighted in bold and a short description of the substitution is provided where required. [file 1745-6150-8-29-S2.docx]

| **Post-translational modification** | **Amino acid** | **Notes** |
| --- | --- | --- |
| Phosphorylation (target amino acids: S, T, Y) | S21[1, 2], T22[2], T23[2], T24[2], Y29[2-24], S76[2], T82[2], Y85[1, 2, 11, 18], Y86[1, 2, 18, 20, 21, 25], T88[1, 2], S128[2], Y141[2, 4-9, 11-15, 17-21, 23, 26-31], T142[2, 12], S157[2], T158[2], Y162[2, 32], S163[2, 32], Y167[2], S175[2], **T176**[2], Y177[2, 6], S205[33], **T234**[2], Y254[2, 20, 27, 34], T261[2], T269[2], T287[2], S300[35], Y357[2], **S358**[33], S383[2], S414[36], Y418[2], T432[10, 26, 36, 37], T452[2], S454[26] | Phosphorylation of **T176** is eEF1A1-specific; present as A176 in eEF1A2.  Phosphorylation of **T234** is eEF1A2-specific; present as C234 in eEF1A1.  Phosphorylation of **S358** is eEF1A2-specific; present as A358 in eEF1A1. |
| Acetylation (target amino acid: K) | K30[2], K36[2], K41[2, 38, 39], K44[2, 39], K55[39], K79[2, 40], K84[2], K146[2, 39], K165[2, 38, 41], K172[2, 38-40], K179[2, 38, 39, 41], K180[2], K212[2], K244[2], K255[2, 38, 39], **K273**[2, 38], K318[39, 42], K392[2, 38, 39], K395[2, 39], K408[2], K439[2, 38, 39], K450[2], K453[2], K457[2], K460[2] | Acetylation of **K273** is eEF1A1-specific; present as R273 in eEF1A2. |
| Methylation (target amino acids: K, R) | K36[43], K55[43, 44] (m1[2, 45], m2[2, 46, 47], m3[2]), K79[43] (m1[2], m2[2], m3[2, 46]), K84 (m1[2], m2[2]), K154 (m1[2]), K165[43, 44] (m1[2, 46], m2[2, 46], m3[2]), R166 (m1[2], m2[2]), K318[43] (m1[2], m2[2]), R382 (m1[2]) | Specific methylation modifications (m1 = mono-, m2 = bi-, m3 = tri-) are provided where information available. |
| Ubiquitination (target amino acid: K) | K41[2, 48-50], K44[2, 48, 50, 51], K84[2, 51], K129[2], K146[2, 48, 49, 51], K154[2, 48, 51], K165[2, 48, 51], K172[2, 48-52], K179[2, 51], K180[2, 48, 51], K212[2], K219[2, 48, 51], K244[2, 52], K255[2, 48-53], **K273**[2, 48, 49, 51, 52], K290[2], K318[2, 51], K385[2, 51], K386[2, 51], K392[2, 48-51], K395[2, 48, 49], K408[2, 48, 51], K439[2, 48-51], K444[2, 51], K450[2, 51] | Ubiquitination of **K273** is eEF1A1-specific; present as R273 in eEF1A2. |
| Glycerylphosphorylethanolamination (target amino acid: E) | E301[43, 54], E374[43, 54] | Study undertaken in eEF1A1 |
| *S*-nitrosylation (target amino acid: C) | **C234**[55], C411[56] | Study undertaken in eEF1A1. *S*-nitrosylation of **C234** is eEF1A1-specific; present as T234 in eEF1A1. |
| *S*-glutathionylation (target amino acid: C) | C411[57] | Study undertaken in eEF1A1 |
| Mono-*O*-glucosylation (target amino acids: S, T, Y) | S53[58] | Study undertaken in eEF1A |
| Carbonylation (target amino acids: mainly K, R, P, T) | Peptides K79-R96[59], K165-K172[59] | Study undertaken in eEF1A1 |
| *S*-modified by prostaglandin (target amino acid: C) | No specific site mentioned in main text[60] | Study undertaken in eEF1A1 |

**Additional file 2: Table of post-translational modifications (PTMs) in eEF1A1 and eEF1A2.** All experimentally derived, curated PTMs in the eukaryotic translation elongation factors 1A1 and 1A2 from human, mouse, rat, and rabbit are provided with corresponding references. The list derives from curated information in the PhosphoSitePlus (www.phosphosite.org; accessed August 2013) and UniProt databases [2, 44], literature involving specific PTM studies in eEF1As, and from both low-throughput experimental methods and high-throughput tandem mass spectrometry. Where data were obtained from mass spectrometric studies from MS assignments from the Cell Signaling Technology research group, the curated results from the PhosphoSitePlus team are listed and referenced. High probability sites represented by five or more references in the PhosphoSitePlus database or those confirmed by experimentation are highlighted in blue (phosphorylation: 22/36; acetylation: 11/25; methylation: 5/9; ubiquitination: 23/25 have five or more citations assigned to them in PhosphoSitePlus); these are mapped on the surface of the 3-D model of eEF1A1 in Additional file 4. Amino acid substitutions that are specific to eEF1A1 or eEF1A2 and that impact on PTMs are highlighted in bold and a short description of the substitution is provided where required.

**References:**

1. Sanges C, Scheuermann C, Zahedi RP, Sickmann A, Lamberti A, Migliaccio N, Baljuls A, Marra M, Zappavigna S, Reinders J, et al: **Raf kinases mediate the phosphorylation of eukaryotic translation elongation factor 1A and regulate its stability in eukaryotic cells.** *Cell Death Dis* 2012, **3:**e276.

2. Hornbeck PV, Kornhauser JM, Tkachev S, Zhang B, Skrzypek E, Murray B, Latham V, Sullivan M: **PhosphoSitePlus: a comprehensive resource for investigating the structure and function of experimentally determined post-translational modifications in man and mouse.** *Nucleic Acids Res* 2012, **40:**D261-270.

3. Pighi C, Gu TL, Dalai I, Barbi S, Parolini C, Bertolaso A, Pedron S, Parisi A, Ren J, Cecconi D, et al: **Phospho-proteomic analysis of mantle cell lymphoma cells suggests a pro-survival role of B-cell receptor signaling.** *Cell Oncol (Dordr)* 2011, **34:**141-153.

4. Bergstrom Lind S, Artemenko KA, Elfineh L, Mayrhofer C, Zubarev RA, Bergquist J, Pettersson U: **Toward a comprehensive characterization of the phosphotyrosine proteome.** *Cell Signal* 2011, **23:**1387-1395.

5. Artemenko KA, Bergstrom Lind S, Elfineh L, Mayrhofer C, Zubarev RA, Bergquist J, Pettersson U: **Optimization of immunoaffinity enrichment and detection: toward a comprehensive characterization of the phosphotyrosine proteome of K562 cells by liquid chromatography-mass spectrometry.** *Analyst* 2011, **136:**1971-1978.

6. Gu TL, Deng X, Huang F, Tucker M, Crosby K, Rimkunas V, Wang Y, Deng G, Zhu L, Tan Z, et al: **Survey of tyrosine kinase signaling reveals ROS kinase fusions in human cholangiocarcinoma.** *PLoS One* 2011, **6:**e15640.

7. Knowlton ML, Selfors LM, Wrobel CN, Gu TL, Ballif BA, Gygi SP, Polakiewicz R, Brugge JS: **Profiling Y561-dependent and -independent substrates of CSF-1R in epithelial cells.** *PLoS One* 2010, **5:**e13587.

8. Bonnette PC, Robinson BS, Silva JC, Stokes MP, Brosius AD, Baumann A, Buckbinder L: **Phosphoproteomic characterization of PYK2 signaling pathways involved in osteogenesis.** *J Proteomics* 2010, **73:**1306-1320.

9. Gu TL, Cherry J, Tucker M, Wu J, Reeves C, Polakiewicz RD: **Identification of activated Tnk1 kinase in Hodgkin's lymphoma.** *Leukemia* 2010, **24:**861-865.

10. Olsen JV, Vermeulen M, Santamaria A, Kumar C, Miller ML, Jensen LJ, Gnad F, Cox J, Jensen TS, Nigg EA, et al: **Quantitative phosphoproteomics reveals widespread full phosphorylation site occupancy during mitosis.** *Sci Signal* 2010, **3:**ra3.

11. Moritz A, Li Y, Guo A, Villen J, Wang Y, MacNeill J, Kornhauser J, Sprott K, Zhou J, Possemato A, et al: **Akt-RSK-S6 kinase signaling networks activated by oncogenic receptor tyrosine kinases.** *Sci Signal* 2010, **3:**ra64.

12. St-Germain JR, Taylor P, Tong J, Jin LL, Nikolic A, Stewart, II, Ewing RM, Dharsee M, Li Z, Trudel S, Moran MF: **Multiple myeloma phosphotyrosine proteomic profile associated with FGFR3 expression, ligand activation, and drug inhibition.** *Proc Natl Acad Sci U S A* 2009, **106:**20127-20132.

13. Nguyen V, Cao L, Lin JT, Hung N, Ritz A, Yu K, Jianu R, Ulin SP, Raphael BJ, Laidlaw DH, et al: **A new approach for quantitative phosphoproteomic dissection of signaling pathways applied to T cell receptor activation.** *Mol Cell Proteomics* 2009, **8:**2418-2431.

14. Choudhary C, Olsen JV, Brandts C, Cox J, Reddy PN, Bohmer FD, Gerke V, Schmidt-Arras DE, Berdel WE, Muller-Tidow C, et al: **Mislocalized activation of oncogenic RTKs switches downstream signaling outcomes.** *Mol Cell* 2009, **36:**326-339.

15. Heibeck TH, Ding SJ, Opresko LK, Zhao R, Schepmoes AA, Yang F, Tolmachev AV, Monroe ME, Camp DG, 2nd, Smith RD, et al: **An extensive survey of tyrosine phosphorylation revealing new sites in human mammary epithelial cells.** *J Proteome Res* 2009, **8:**3852-3861.

16. Mayya V, Lundgren DH, Hwang SI, Rezaul K, Wu L, Eng JK, Rodionov V, Han DK: **Quantitative phosphoproteomic analysis of T cell receptor signaling reveals system-wide modulation of protein-protein interactions.** *Sci Signal* 2009, **2:**ra46.

17. Luo W, Slebos RJ, Hill S, Li M, Brabek J, Amanchy R, Chaerkady R, Pandey A, Ham AJ, Hanks SK: **Global impact of oncogenic Src on a phosphotyrosine proteome.** *J Proteome Res* 2008, **7:**3447-3460.

18. Guo A, Villen J, Kornhauser J, Lee KA, Stokes MP, Rikova K, Possemato A, Nardone J, Innocenti G, Wetzel R, et al: **Signaling networks assembled by oncogenic EGFR and c-Met.** *Proc Natl Acad Sci U S A* 2008, **105:**692-697.

19. Ballif BA, Carey GR, Sunyaev SR, Gygi SP: **Large-scale identification and evolution indexing of tyrosine phosphorylation sites from murine brain.** *J Proteome Res* 2008, **7:**311-318.

20. Rikova K, Guo A, Zeng Q, Possemato A, Yu J, Haack H, Nardone J, Lee K, Reeves C, Li Y, et al: **Global survey of phosphotyrosine signaling identifies oncogenic kinases in lung cancer.** *Cell* 2007, **131:**1190-1203.

21. Gu TL, Mercher T, Tyner JW, Goss VL, Walters DK, Cornejo MG, Reeves C, Popova L, Lee K, Heinrich MC, et al: **A novel fusion of RBM6 to CSF1R in acute megakaryoblastic leukemia.** *Blood* 2007, **110:**323-333.

22. Walters DK, Goss VL, Stoffregen EP, Gu TL, Lee K, Nardone J, McGreevey L, Heinrich MC, Deininger MW, Polakiewicz R, Druker BJ: **Phosphoproteomic analysis of AML cell lines identifies leukemic oncogenes.** *Leuk Res* 2006, **30:**1097-1104.

23. Goss VL, Lee KA, Moritz A, Nardone J, Spek EJ, MacNeill J, Rush J, Comb MJ, Polakiewicz RD: **A common phosphotyrosine signature for the Bcr-Abl kinase.** *Blood* 2006, **107:**4888-4897.

24. Rush J, Moritz A, Lee KA, Guo A, Goss VL, Spek EJ, Zhang H, Zha XM, Polakiewicz RD, Comb MJ: **Immunoaffinity profiling of tyrosine phosphorylation in cancer cells.** *Nat Biotechnol* 2005, **23:**94-101.

25. Kim JE, White FM: **Quantitative analysis of phosphotyrosine signaling networks triggered by CD3 and CD28 costimulation in Jurkat cells.** *J Immunol* 2006, **176:**2833-2843.

26. Huttlin EL, Jedrychowski MP, Elias JE, Goswami T, Rad R, Beausoleil SA, Villen J, Haas W, Sowa ME, Gygi SP: **A tissue-specific atlas of mouse protein phosphorylation and expression.** *Cell* 2010, **143:**1174-1189.

27. Iliuk AB, Martin VA, Alicie BM, Geahlen RL, Tao WA: **In-depth analyses of kinase-dependent tyrosine phosphoproteomes based on metal ion-functionalized soluble nanopolymers.** *Mol Cell Proteomics* 2010, **9:**2162-2172.

28. Wisniewski JR, Nagaraj N, Zougman A, Gnad F, Mann M: **Brain phosphoproteome obtained by a FASP-based method reveals plasma membrane protein topology.** *J Proteome Res* 2010, **9:**3280-3289.

29. Cao L, Yu K, Banh C, Nguyen V, Ritz A, Raphael BJ, Kawakami Y, Kawakami T, Salomon AR: **Quantitative time-resolved phosphoproteomic analysis of mast cell signaling.** *J Immunol* 2007, **179:**5864-5876.

30. Wolf-Yadlin A, Kumar N, Zhang Y, Hautaniemi S, Zaman M, Kim HD, Grantcharova V, Lauffenburger DA, White FM: **Effects of HER2 overexpression on cell signaling networks governing proliferation and migration.** *Mol Syst Biol* 2006, **2:**54.

31. Schmelzle K, Kane S, Gridley S, Lienhard GE, White FM: **Temporal dynamics of tyrosine phosphorylation in insulin signaling.** *Diabetes* 2006, **55:**2171-2179.

32. Molina H, Horn DM, Tang N, Mathivanan S, Pandey A: **Global proteomic profiling of phosphopeptides using electron transfer dissociation tandem mass spectrometry.** *Proc Natl Acad Sci U S A* 2007, **104:**2199-2204.

33. Gandin V, Gutierrez GJ, Brill LM, Varsano T, Feng Y, Aza-Blanc P, Au Q, McLaughlan S, Ferreira TA, Alain T, et al: **Degradation of Newly Synthesized Polypeptides by Ribosome-Associated RACK1/c-Jun N-Terminal Kinase/Eukaryotic Elongation Factor 1A2 Complex.** *Mol Cell Biol* 2013, **33:**2510-2526.

34. Daub H, Olsen JV, Bairlein M, Gnad F, Oppermann FS, Korner R, Greff Z, Keri G, Stemmann O, Mann M: **Kinase-selective enrichment enables quantitative phosphoproteomics of the kinome across the cell cycle.** *Mol Cell* 2008, **31:**438-448.

35. Lin KW, Yakymovych I, Jia M, Yakymovych M, Souchelnytskyi S: **Phosphorylation of eEF1A1 at Ser300 by TbetaR-I results in inhibition of mRNA translation.** *Curr Biol* 2010, **20:**1615-1625.

36. Old WM, Shabb JB, Houel S, Wang H, Couts KL, Yen CY, Litman ES, Croy CH, Meyer-Arendt K, Miranda JG, et al: **Functional proteomics identifies targets of phosphorylation by B-Raf signaling in melanoma.** *Mol Cell* 2009, **34:**115-131.

37. Eckhardt K, Troger J, Reissmann J, Katschinski DM, Wagner KF, Stengel P, Paasch U, Hunziker P, Borter E, Barth S, et al: **Male germ cell expression of the PAS domain kinase PASKIN and its novel target eukaryotic translation elongation factor eEF1A1.** *Cellular Physiology and Biochemistry* 2007, **20:**227-240.

38. Simon GM, Cheng J, Gordon JI: **Quantitative assessment of the impact of the gut microbiota on lysine epsilon-acetylation of host proteins using gnotobiotic mice.** *Proc Natl Acad Sci U S A* 2012, **109:**11133-11138.

39. Choudhary C, Kumar C, Gnad F, Nielsen ML, Rehman M, Walther TC, Olsen JV, Mann M: **Lysine acetylation targets protein complexes and co-regulates major cellular functions.** *Science* 2009, **325:**834-840.

40. Zhao S, Xu W, Jiang W, Yu W, Lin Y, Zhang T, Yao J, Zhou L, Zeng Y, Li H, et al: **Regulation of cellular metabolism by protein lysine acetylation.** *Science* 2010, **327:**1000-1004.

41. Schwer B, Eckersdorff M, Li Y, Silva JC, Fermin D, Kurtev MV, Giallourakis C, Comb MJ, Alt FW, Lombard DB: **Calorie restriction alters mitochondrial protein acetylation.** *Aging Cell* 2009, **8:**604-606.

42. Kim SC, Sprung R, Chen Y, Xu Y, Ball H, Pei J, Cheng T, Kho Y, Xiao H, Xiao L, et al: **Substrate and functional diversity of lysine acetylation revealed by a proteomics survey.** *Mol Cell* 2006, **23:**607-618.

43. Dever TE, Costello CE, Owens CL, Rosenberry TL, Merrick WC: **Location of seven post-translational modifications in rabbit elongation factor 1 alpha including dimethyllysine, trimethyllysine, and glycerylphosphorylethanolamine.** *J Biol Chem* 1989, **264:**20518-20525.

44. UniProtConsortium: **Update on activities at the Universal Protein Resource (UniProt) in 2013.** *Nucleic Acids Res* 2013, **41:**D43-47.

45. Jung SY, Li Y, Wang Y, Chen Y, Zhao Y, Qin J: **Complications in the assignment of 14 and 28 Da mass shift detected by mass spectrometry as in vivo methylation from endogenous proteins.** *Anal Chem* 2008, **80:**1721-1729.

46. Uhlmann T, Geoghegan VL, Thomas B, Ridlova G, Trudgian DC, Acuto O: **A method for large-scale identification of protein arginine methylation.** *Mol Cell Proteomics* 2012, **11:**1489-1499.

47. Grant JE, Hu J, Liu T, Jain MR, Elkabes S, Li H: **Post-translational modifications in the rat lumbar spinal cord in experimental autoimmune encephalomyelitis.** *J Proteome Res* 2007, **6:**2786-2791.

48. Wagner SA, Beli P, Weinert BT, Scholz C, Kelstrup CD, Young C, Nielsen ML, Olsen JV, Brakebusch C, Choudhary C: **Proteomic analyses reveal divergent ubiquitylation site patterns in murine tissues.** *Mol Cell Proteomics* 2012, **11:**1578-1585.

49. Wagner SA, Beli P, Weinert BT, Nielsen ML, Cox J, Mann M, Choudhary C: **A proteome-wide, quantitative survey of in vivo ubiquitylation sites reveals widespread regulatory roles.** *Mol Cell Proteomics* 2011, **10:**M111 013284.

50. Danielsen JM, Sylvestersen KB, Bekker-Jensen S, Szklarczyk D, Poulsen JW, Horn H, Jensen LJ, Mailand N, Nielsen ML: **Mass spectrometric analysis of lysine ubiquitylation reveals promiscuity at site level.** *Mol Cell Proteomics* 2011, **10:**M110 003590.

51. Kim W, Bennett EJ, Huttlin EL, Guo A, Li J, Possemato A, Sowa ME, Rad R, Rush J, Comb MJ, et al: **Systematic and quantitative assessment of the ubiquitin-modified proteome.** *Mol Cell* 2011, **44:**325-340.

52. Xu G, Paige JS, Jaffrey SR: **Global analysis of lysine ubiquitination by ubiquitin remnant immunoaffinity profiling.** *Nat Biotechnol* 2010, **28:**868-873.

53. Shi Y, Chan DW, Jung SY, Malovannaya A, Wang Y, Qin J: **A data set of human endogenous protein ubiquitination sites.** *Mol Cell Proteomics* 2011, **10:**M110 002089.

54. Rosenberry TL, Krall JA, Dever TE, Haas R, Louvard D, Merrick WC: **Biosynthetic incorporation of [3H]ethanolamine into protein synthesis elongation factor 1 alpha reveals a new post-translational protein modification.** *J Biol Chem* 1989, **264:**7096-7099.

55. Greco TM, Hodara R, Parastatidis I, Heijnen HF, Dennehy MK, Liebler DC, Ischiropoulos H: **Identification of S-nitrosylation motifs by site-specific mapping of the S-nitrosocysteine proteome in human vascular smooth muscle cells.** *Proc Natl Acad Sci U S A* 2006, **103:**7420-7425.

56. Lam YW, Yuan Y, Isaac J, Babu CV, Meller J, Ho SM: **Comprehensive identification and modified-site mapping of S-nitrosylated targets in prostate epithelial cells.** *PLoS One* 2010, **5:**e9075.

57. Hamnell-Pamment Y, Lind C, Palmberg C, Bergman T, Cotgreave IA: **Determination of site-specificity of S-glutathionylated cellular proteins.** *Biochem Biophys Res Commun* 2005, **332:**362-369.

58. Belyi Y, Stahl M, Sovkova I, Kaden P, Luy B, Aktories K: **Region of elongation factor 1A1 involved in substrate recognition by Legionella pneumophila glucosyltransferase Lgt1: identification of Lgt1 as a retaining glucosyltransferase.** *J Biol Chem* 2009, **284:**20167-20174.

59. Grimsrud PA, Picklo MJ, Sr., Griffin TJ, Bernlohr DA: **Carbonylation of adipose proteins in obesity and insulin resistance: identification of adipocyte fatty acid-binding protein as a cellular target of 4-hydroxynonenal.** *Mol Cell Proteomics* 2007, **6:**624-637.

60. Gharbi S, Garzon B, Gayarre J, Timms J, Perez-Sala D: **Study of protein targets for covalent modification by the antitumoral and anti-inflammatory prostaglandin PGA1: focus on vimentin.** *J Mass Spectrom* 2007, **42:**1474-1484.
