# Supplementary material for: Highly homologous eEF1A1 and eEF1A2 exhibit differential post-translational modification with significant enrichment around localised sites of sequence variation
Source: Biol Direct. 2013 Nov 13;8:29. doi: 10.1186/1745-6150-8-29 (PMC3868327; doi:10.1186/1745-6150-8-29)
Supplement: Additional file 3 — Post-translational modifications (PTMs) in structural proximity to sequence variants between eEF1A1 and eEF1A2. A total of 42 out of 67 (62.7%) PTM sites are located structurally-proximal to a variant amino acid residue between eEF1A1 and eEF1A2; assessed using a 5-Å sphere radius probe measurement around each variant amino acid on the 3-D model of human eEF1A1 [5] under PyMol (http://www.pymol.org; The PyMOL Molecular Graphics System, Version 0.99 Schrödinger, LLC). Those variants that are altered between human eEF1A1 and eEF1A2 and are also sites of post-translational modification are highlighted in bold. Note; there are an additional seven PTM sites located in the unstructured C-terminus of the proteins that harbour four additional sites of sequence variation between eEF1A1 and eEF1A2, not shown. Out of the 42 PTMs located proximal to the variant amino acid residues, a total of 33 are surface exposed at or proximal to the two surface clusters of sequence variation (a total of 20 for cluster 1, and 13 for cluster 2). Twelve of these cluster-proximal PTM sites are modified more than once. Symbols added at the end of each PTM in the table indicate: –p: phosphorylation; -a: acetylation; -m: methylation; -u: ubiquitination; -e: glycerylphosphorylethanolamination; -n: S-nitrosylation; -t: S-glutathionylation. [file 1745-6150-8-29-S3.docx]

| **Variant amino acid residue number** | **“Conserved” or “Variable” surface of protein?** | **Cluster?** | **Post-translational modifications within 5 Å of variant residue** |
| --- | --- | --- | --- |
| S83T | Variable | Cluster 1 | T82-p; K84-a/m/u; Y85-p |
| V87I | Buried | - | K79-a/m; T82-p; Y85-p; Y86-p; T88-p |
| P161A | Variable/Conserved | Cluster 1 | T158-p; Y162-p; S163-p; K165-a/m/u; R166-m; Y167-p; S205-p |
| Q164E | Variable | Cluster 1 | T158-p; Y162-p; S163-p; K165-a/m/u; R166-m; Y167-p; K212-a/u |
| E168D | Variable | Cluster 1 | T158-p; S163-p; K165-a/m/u; R166-m; Y167-p; K172-a/u; S175-p |
| **T176A** | Variable | Cluster 1 | K172-a/u; S175-p; **T176-p**; Y177-p; K179-a/u; K180-a/u |
| D186A | Variable | Cluster 1 | - |
| A189P | Buried | - | Y167-p; **C234T-n/p** |
| N197H | Conserved | - | K55-a/m; Y162-p; S205-p |
| A206P | Variable/Conserved | Cluster 1 | Y162-p; S205-p |
| T217E | Variable | Cluster 1 | K219-u |
| D220E | Variable | Cluster 1 | K219-u |
| T226V | Variable | Cluster 1 | T23-p; S205-p; K212-a/u |
| T227S | Variable | Cluster 1 | T82-p; S205-p; **C234T-n/p** |
| **C234T** | Variable | Cluster 1 | K84-a/m/u; Y85-p; K219-u; **C234T-n/p** |
| V271I | Variable | Cluster 2 | K244-a/u; T269-p; **K273R-a/u**; S300-p; E301-e |
| **K273R** | Variable | Cluster 2 | **K273R-a/u**; K290-u; S300-p; E301-e |
| V285I |  | Cluster 2 | T287-p; K290-u |
| V320I | Conserved | - | T261-p; K318-a/m/u |
| A326C | Buried | - | - |
| N331S | Variable | Cluster 2 | - |
| M335Q | Variable | Cluster 2 | C411-n/t |
| G339Q | Variable | Cluster 2 | K439-a/u |
| A342S | Buried | - | K439-a/u |
| **A358S** | Conserved | - | Y357-p; **A358S-p** |
| L361I | Buried | - | - |
| G390N | Variable/Conserved | Cluster 2 | E374-e; K392-a/u |
| F393S | Conserved | - | K392-a/u; K395-a/u |
| D403E | Variable | Cluster 2 | E374-e |
| D417Q | Variable | Cluster 2 | K244-a/u; T269-p; S414-p; Y418-p |
| A440N | Variable | Cluster 2 | S414-p; K439-a/u |
| D442E | Variable | Cluster 2 | - |

**Additional file 3: Post-translational modifications (PTMs) in structural proximity to sequence variants between eEF1A1 and eEF1A2.** A total of 42 out of 67 (62.7%) PTM sites are located structurally-proximal to a variant amino acid residue between eEF1A1 and eEF1A2; assessed using a 5-Å sphere radius probe measurement around each variant amino acid on the 3-D model of human eEF1A1 [5] under PyMol (www.pymol.org; The PyMOL Molecular Graphics System, Version 0.99 Schrödinger, LLC). Those variants that are altered between human eEF1A1 and eEF1A2 and are also sites of post-translational modification are highlighted in bold. Note; there are an additional seven PTM sites located in the unstructured C-terminus of the proteins that harbour four additional sites of sequence variation between eEF1A1 and eEF1A2, not shown. Out of the 42 PTMs located proximal to the variant amino acid residues, a total of 33 are surface exposed at or proximal to the two surface clusters of sequence variation (a total of 20 for cluster 1, and 13 for cluster 2). Twelve of these cluster-proximal PTM sites are modified more than once. Symbols added at the end of each PTM in the table indicate: –p: phosphorylation; -a: acetylation; -m: methylation; -u: ubiquitination; -e: glycerylphosphorylethanolamination; -n: *S*-nitrosylation; -t: *S*-glutathionylation.

**References:**

1. Soares DC, Barlow PN, Newbery HJ, Porteous DJ, Abbott CM: **Structural models of human eEF1A1 and eEF1A2 reveal two distinct surface clusters of sequence variation and potential differences in phosphorylation.** *PLoS One* 2009, **4:**e6315.
