# Supplementary material for: Highly homologous eEF1A1 and eEF1A2 exhibit differential post-translational modification with significant enrichment around localised sites of sequence variation
Source: Biol Direct. 2013 Nov 13;8:29. doi: 10.1186/1745-6150-8-29 (PMC3868327; doi:10.1186/1745-6150-8-29)
Supplement: Additional file 4 — Post-translational modifications (PTMs) by strength of evidence mapped on surface of eEF1A1. (A) All experimentally derived, curated PTMs in the eukaryotic translation elongation factors 1A1 and 1A2 from human, mouse, rat, and rabbit are shown mapped on the 3-D model of eEF1A1 as in Figure 1 in the main text; refer to legend therein. (B) Only those sites represented by five or more citations in the PhosphoSitePlus database or those confirmed by site-specific experiment in the literature are shown on the 3-D model of human eEF1A1. PTM residues that do not meet this criterion are labelled in the upper frame (A) for comparison. The vast majority of surface exposed residues that harbour PTMs are retained. [file 1745-6150-8-29-S4.docx]

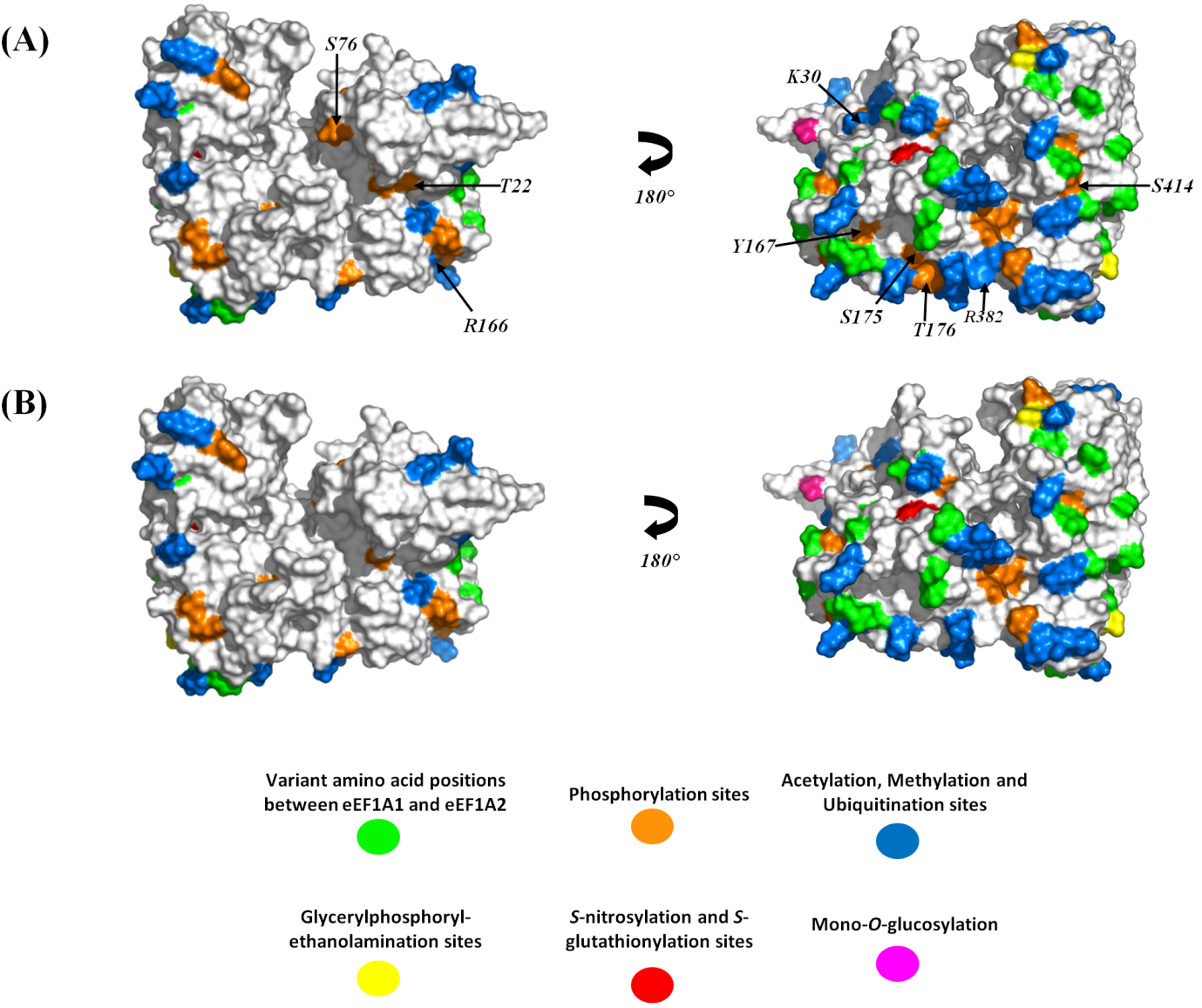


**Additional file 4: Post-translational modifications (PTMs) by strength of evidence mapped on surface of eEF1A1. (A)** All experimentally derived, curated PTMs in the eukaryotic translation elongation factors 1A1 and 1A2 from human, mouse, rat, and rabbit are shown mapped on the 3-D model of eEF1A1 as in Figure 1 in the main text; refer to legend therein. **(B)** Only those sites represented by five or more citations in the PhosphoSitePlus database or those confirmed by site-specific experiment in the literature are shown on the 3-D model of human eEF1A1. PTM residues that do not meet this criterion are labelled in the upper frame (A) for comparison. The vast majority of surface exposed residues that harbour PTMs are retained.
